# Industry Payments to Nephrologists in the United States

**DOI:** 10.7759/cureus.17057

**Published:** 2021-08-10

**Authors:** Abhinandan R Pakanati, Karthik Kovvuru, Vaishali Thombre, Swetha Rani Kanduri, Krishna Nalleballe, Saritha Ranabothu

**Affiliations:** 1 Nephrology, The Kidney Clinic, LLC, Snellville, USA; 2 Nephrology, Ochsner Medical Center, New Orleans, USA; 3 Biostatistics and Epidemiology, University of Arkansas for Medical Sciences, Little Rock, USA; 4 Neurology, University of Arkansas for Medical Sciences, Little Rock, USA; 5 Pediatrics, University of Arkansas for Medical Sciences, Little Rock, USA

**Keywords:** health economics, industry payment, open payment program, physician beneficiaries, sunshine act, nephrology

## Abstract

Background

Industry payments to physicians raise concerns about conflicts of interest that have the potential to impact patient care. In this study, we explored nonresearch and nonownership payments from industry to nephrologists to identify trends in compensation.

Methodology

Using data from the Centers for Medicare and Medicaid Services (CMS), we explored financial relationships between industry and US nephrologists from 2014 to 2018. We analyzed payment characteristics including payment categories, payment distribution among physicians, regional trends, and biomedical manufacturers.

Results

In this retrospective study, a total of $75,174,999 was paid to nephrologists in the United States during the study period (i.e., 2014-2018). The number of board-certified nephrologists receiving payment from the industry increased from 11,642 in 2014 to 13,297 in 2018. Among board-certified nephrologists, 56% to 63% received industry payments during the study period. The total payments to nephrologists increased from $13,113,512 in 2014 to $16,467,945 in 2017, with consulting fees (24%) and compensation for services other than consulting (35%) being the highest-paid categories. The top 10% of physician beneficiaries collected 90% of the total industry payments.

Conclusions

A small proportion of US nephrologists consistently received the majority of industry payments, the value of which grew over the study period.

## Introduction

Physicians often collaborate with organizations in the healthcare industry. Approximately 94% of physicians in the United States report some relationship with the industry which ranges from receiving food to being paid for various services [1]. Even though such collaborations can encourage research and patient care, they introduce potential conflicts of interest. For example, receiving industry-sponsored meals has been associated with increased prescription of brand-name medications [2,3]. Industry-sponsored research, compared to nonindustry-sponsored research, shows that these practices have more favorable results and less evidence of harm [4].

Due to concerns about the potential detrimental effects of physician-industry financial relationships, the Centers for Medicare and Medicaid Services (CMS) Open Payments Program (OPP) was initiated by the Physician Payments Sunshine Act of 2009 [5]. CMS OPP mandates public reporting of industry payments made to physicians, teaching hospitals, group purchasing organizations, and medical product manufacturers [6]. Such information about eligible payments has been available through the CMS OPP website since August 2013. Physician­-industry financial relationships have been reported in several clinical specialties [7,8], and this study reports the trends and details of such payments in the field of nephrology.

## Materials and methods

Data sources

Data on industry payments to nephrologists reported from January 1, 2014, through December 31, 2018, were extracted from the CMS OPP database, and a retrospective analysis was performed. Only general payments were analyzed, and research and partnership payments were excluded. The general payments were divided into nine subcategories (Table 1). The database record for each payment carries a unique physician profile identification, recipient state, sponsor, and related product. Each payment to each physician has a separate entry, even if the physician received multiple payments.

**Table 1:** Nine subcategories of the general payment. Adopted from https://www.cms.gov/OpenPayments/About/Natures-of-Payment.

Subcategory	Definition
Consulting fee	Payments made to physicians for advice and expertise on a particular medical product or treatment, typically provided under a written agreement and in response to a particular business need. These payments vary depending on the experience of the physician being consulted
Education	This category generally includes payments or transfers of value for classes, activities, programs, or events that involve the imparting or acquiring of particular knowledge or skills, such as those used for a profession. This category can include things like textbooks and medical journal articles
Entertainment	Attendance at recreational, cultural, sporting, or other events that would generally have a cost
Gift	A general category that often includes anything provided to a physician or teaching hospital that does not fit into another category
Grant	Payments to a physician or teaching hospital in support of a specific cause or activity
Honoraria	Similar to consulting fees, but generally reserved for a one-time, short-duration activity. Also distinguishable in that they are generally provided for services that custom prohibits a price from being set
Travel and lodging	Travel and lodging
Food and beverage	Food and beverage
Compensation for services other than consulting	Payments made to physicians for speaking, training, and education engagements that are not for continuing education

Data analysis

To obtain the total amount paid to each nephrologist, individual payments to each unique physician profile identification were added. From these calculations, 10% of the nephrologists who received the highest amount of industry payments each year were identified. For each year, the sum of all payments received by the top 10% of nephrologists was calculated as a proportion of the total amount of industry payments.

Using the total number of nephrologists in the United States as a denominator, we calculated the proportion of nephrologists who received industry payments. The mean, median, minimum, maximum, and 90th percentile were calculated for these payments. To explore the distribution of payments among US nephrologists, we calculated the Gini index (a statistical measure of dispersion) for physicians who received any payment each year. The Gini index ranges from 0 (all physicians received an equal number of payments) to 1 (one physician received all the payments).

## Results

Results of this retrospective study revealed that the industry paid a total of $75,174,999 to US nephrologists during the study period 2014-2018 (Table 2). Total payments to nephrologists increased slowly and persistently from 2014 to 2017, with a slight downward trend noted in 2018. The number of board-certified nephrologists increased from 11,642 in 2014 to 13,297 in 2018; 56-63% of board-certified nephrologists received industry payments during the study period. Each year, about 7,300 nephrologists received payments, and the top 10% received 90% of the total payments. Mean payments ($1,795-$2,227) were substantially higher than the median payments ($145-$184) (Table 3). The Gini index ranged from 0.90 to 0.92 (Table 3).

**Table 2:** Summary of payments from industry to board-certified nephrologists (2014–2018).

Year	General payment	Percentage	Research payment	Percentage	Ownership payment	Percentage	Total
2014	$13,113,512	68%	$1,540,777	8%	$4,585,718	24%	$19,240,007
2015	$14,659,590	69%	$2,951,263	14%	$3,718,113	17%	$21,328,967
2016	$15,504,983	79%	$2,748,154	14%	$1,263,222	6%	$19,516,359
2017	$16,467,945	64%	$1,617,353	6%	$7,669,002	30%	$25,754,300
2018	$15,428,969	80%	$241,579	1%	$3,624,535	19%	$19,295,082
Total	$75,174,999	72%	$9,099,126	9.7%	$20,860,590	20.7%	$105,134,715

**Table 3:** Characteristics of general payments to nephrologists (2014–2018).

Payment descriptor	2014	2015	2016	2017	2018
Board-certified nephrologists	11,642	12,129	12,567	12,944	13,297
Board-certified nephrologists receiving payment (% of total)	7,304 (63%)	7,199 (59%)	7,308 (58%)	7,394 (57%)	7,393 (56%)
Total value of payments	$13,113,512	$14,659,590	$15,504,983	$16,467,945	$15,428,969
Mean payment	$1,795	$2,036	$2,122	$2,227	$2,087
Standard deviation	$18,945	$14,620	$14,504	$14,358	$13,501
Median payment	$147	$145	$156	$184	$179
Interquartile range	$296	$323	$345	$415	$415
Gini index	0.92	0.91	0.92	0.91	0.90
Total amount received by the top 10% of nephrologists	$11,848,107	$13,294,746	$14,045,348	$14,802,345	$13,787,134
Percentage of total value of payments	90%	91%	91%	90%	89%
Maximum payment	$1,091,314	$680,304	$459,664	$527,635	$648,182
Top 10 physician payments	$3,863,967	$2,765,727	$2,850,077	$2,764,036	$2,573,686
Percentage of top 10 physician payments	29%	19%	18%	17%	17%

The three highest payment categories, namely, speaker fee (35%), consulting fee (24%), and travel and lodging (15.6%), were consistently prominent throughout the study period (Table 4). Compensation for continuing medical education remained surprisingly low throughout the study period, but compensation for food and beverage trended linearly upward from 2014 to 2017 ($1,740,090 to $2,369,407). Interestingly, physician payments for royalties or licenses were moderately high in 2014 but trended downward in subsequent years.

**Table 4:** Categories of general payments to nephrologists (2014–2018).

Category	2014	2015	2016	2017	2018	Total
Charitable contribution	$20,000	$1,000	$25,000	-	$30,603	$76,603
Compensation for service	$2,374,739	$5,145,838	$6,185,955	$6,854,196	$5,978,104	$26,538,832
Consulting fee	$3,286,599	$4,135,067	$3,181,342	$3,947,520	$3,844,884	$18,395,411
Current or prospect	-	-	$350	-	-	$350
Education	$205,789	$70,525	$74,054	$199,561	$73,044	$622,973
Entertainment	$2,139	$225	$8,555	$1,816	$94	$12,829
Food and beverage	$1,740,090	$1,873,092	$2,061,513	$2,369,407	$2,314,527	$10,358,629
Gift	$7,818	$5,185	$58,899	$26,975	$8,167	$107,045
Grant	$13,461	$13,275	$66,200	$50,723	$1,167	$144,826
Honoraria	$1,704,677	$355,907	$1,083,312	$435,798	$822,202	$4,401,896
Royalty or license	$1,792,183	$445,715	$337,888	$87,356	$50,072	$2,713,214
Travel and lodging	$1,966,017	$2,613,761	$2,421,915	$2,494,594	$2,306,105	$11,802,392
Total	$13,113,512	$14,659,590	$15,504,983	$16,467,945	$15,428,969	$75,174,999

Veltassa, Acthar, and Auryxia were the drugs most commonly associated with payments to nephrologists in 2018 (Table 5). These drugs consistently ranked high among the drugs associated with payments from 2016 to 2018. Soliris was one of the top drugs associated with the highest payments to nephrologists in 2016, but payments associated with Soliris decreased in 2017 and 2018.

**Table 5:** Leading manufacturers and drugs associated with the highest amounts of payments to board-certified nephrologists (2014–2018). #N/A: not applicable, drug information not provided. *System One hemodialysis machine is a device manufactured by NxStage Medical, Inc. (Lawrence, Massachusetts, USA).

2014		2015		2016		2017		2018	
Drug/Device	Sum of payments	Drug/Device	Sum of payments	Drug/Device	Sum of payments	Drug/Device	Sum of payments	Drug/Device	Sum of payments
N/A^#^	$5,254,268	N/A^#^	$4,105,328	N/A^#^	$2,336,365	N/A^#^	$2,688,686	N/A^#^	$2,435,404
Acthar	$2,184,522	Acthar	$1,672,232	Acthar	$2,100,424	Acthar	$1,674,653	Veltassa	$1,748,937
Soliris	$696,385	Soliris	$1,632,666	Veltassa	$1,344,142	Veltassa	$1,504,282	Acthar	$1,607,383
Sensipar	$564,627	Auryxia	$1,136,523	Soliris	$1,191,279	Velphoro	$986,230	Auryxia	$1,028,136
Invokana	$366,892	Velphoro	$861,981	Velphoro	$815,135	Soliris	$950,499	Parsabiv	$978,166
Samsca	$307,925	Sensipar	$451,259	Auryxia	$687,898	Parsabiv	$925,652	Velphoro	$746,597
Bydureon	$305,322	Fabrazyme	$363,125	Samsca	$598,621	Samsca	$797,734	Jynarque	$668,011
Fabrazyme	$272,039	Invokana	$357,232	Invokana	$590,270	Invokana	$594,591	Soliris	$455,594
Renvela	$259,388	Bydureon	$338,018	Sensipar	$555,172	Tradjenta	$594,269	Invokana	$440,035
Rituxan	$199,044	Uloric	$337,099	Farxiga	$533,527	Farxiga	$444,203	Tradjenta	$369,155
Noncovered	$168,164	Renvela	$329,853	System One*	$432,312	Auryxia	$380,276	Samsca	$316,473

## Discussion

The present study used the CMS OPP database to explore the financial relationships between industry and US nephrologists over a five-year period. Results revealed that total payments increased from 2014 to 2017, which was followed by a downturn in 2018. Although the exact reason for this trend is unclear, it could be related to changes in the industry’s activities due to OPP and the Sunshine Act. However, it is unclear whether underreporting occurred.

Additionally, even though the number of board-certified nephrologists increased during the study period and annual payments to nephrologists increased tremendously, the proportion of nephrologists receiving payments remained relatively stable throughout the study period. Similar to results reported for other subspecialties, such as cardiology and vascular neurology [9,10], we report substantial inequality among nephrologists receiving industry payments. Overall, 90% of the total payments were received by only 10% of the nephrologists. This pattern of skewed payments may be related to industries targeting physicians who are perceived as leaders in the field with the ability to influence the practice patterns of other physicians [11,12]. It is encouraging that most nephrologists did not receive significant payments, so they are not particularly prone to the potential biases introduced by financial relationships with the industry.

Compensation for services, consulting fees, and travel and lodging consistently remained the top payment categories throughout the study period. Categories varied widely among different specialties, with royalties and licensing accounting for the highest-paid category among surgical specialties [13,14]. Consistent with reports from other medical subspecialties, our results showed that payments were the largest for engagements related to noneducational activities [15]. In nephrology, medications remain important categories for industrial payments, which differs from the field of vascular neurology in which payments shifted from pharmacological utilities to medical devices [9].

A few limitations of our study must be noted. Our retrospective analysis is based on information obtained from the CMS OPP database, which could have ambiguities and incomplete records. Potential inaccuracies may result from the retrospective nature of the study and its reliance on self-reported data. Additionally, nephrologists who are not board-certified were excluded. We did not explore research and ownership payments by the industry and their compensation trends. Finally, we could not report the most recent trends (i.e., 2020-2021) because we were limited by data collected through 2018.

## Conclusions

We report that the industry paid the greatest amount of money to a small percentage of nephrologists, which was consistent throughout the study period. Controversy continues to surround financial relationships between industry and physicians, as well as the potential influence of these relationships on research and patient care. Further studies are warranted to help overcome the limitations of the present study and to increase our understanding of financial conflicts of interest and the subsequent impact on patient care.
